# Editorial: Platelets as Players in Neuropathologies: Novel Diagnostic and Therapeutic Targets

**DOI:** 10.3389/fimmu.2021.772352

**Published:** 2021-09-29

**Authors:** Samuel C. Wassmer, Christian Humpel, Jacqueline M. Orian

**Affiliations:** ^1^Department of Infection Biology, London School of Hygiene and Tropical Medicine, London, United Kingdom; ^2^Laboratory for Psychiatry and Experimental Alzheimer’s Research, Medical University Innsbruck, Innsbruck, Austria; ^3^Department of Biochemistry and Genetics, La Trobe Institute for Molecular Science, La Trobe University, Melbourne, VIC, Australia

**Keywords:** platelets, neuroinflammatory diseases, neurodegenerative diseases, biomarkers, therapeutic targets

The revised view of platelets, or thrombocytes, from megakaryocytes-derived cell fragments dedicated to hemostasis, to pivotal elements in inflammation and autoimmunity is now well-documented (1, 2). Over recent decades, unexpected evidence has highlighted the multifaceted functions of these unique cells (3–5):

1. Platelets are anucleate cells of 1.5-3 µm in diameter in humans (0.5-1.0 µm in mice), of high abundance (150-450 x10^3^/µl in humans; 1,000-1,500 x10^3^/µl in mice) and short lifespan (8-9 days in humans; 4-5 days in mice). However, their estimated 3,700 different proteins, relate not only to hemostasis, but to defence, cell-cell communication and the inflammatory response (6, 7).
2. Platelets carry rough endoplasmic reticulum, polyribosomes and stable megakaryocyte-derived mRNA transcripts, selected during thrombopoiesis. Also identified are 284 miRNA species regulating protein expression *via* miRNA-mRNA pairings (8, 9).
3. Platelets are high extracellular vesicle (EV) producers. Platelet-derived microvesicles account for up to 70–90 % of total EV in peripheral blood (10).
4. Platelets exhibit rapid changes of phenotype by acquiring unique mRNA and protein profiles, depending on pathological status (11).

Consequently, platelets instantaneously sense danger signals and respond by recruitment of innate immune cells, triggering an adaptive immune response. In this context, we organized a Research Topic in Frontiers in Immunology with a focus on two themes: (1) similarities between platelets and neurons in expression profile and (2) their potential as biomarkers and therapeutic targets. We gathered five original papers and four reviews on the role of platelets in neuroinflammatory diseases, such as multiple sclerosis (MS) and neurodegenerative/neuropsychiatric disorders, particularly stroke, Alzheimer disease (AD) and Parkinson’s disease (PD).

The review by Leiter and Walker provides an updated overview of current evidence on platelet function and details how platelets are pivotal to immune responses, tissue remodeling and healthy brain function. Significantly, platelets express multiple components regarded as bona fide neuronal proteins, including neurotransmitters for central nervous system intercellular communication, neurogenesis-enhancing molecules, components promoting neuronal plasticity and Alzheimer’s precursor protein (APP) and its metabolite beta-amyloid (Aß). Additionally, there are shared mechanisms between platelets and neurons in neurotransmitter storage and release, secretory pathways and uptake and packaging (Leiter and Walker) (12). Therefore, platelet hyperactivation has major implications in neurodegenerative conditions.

Consequently, the identification of release markers of platelet activation is a major pursuit. Inyushin and colleagues explore the role of systemic platelet-derived APP and Aß peptides, particularly the Aß_1-40_ peptide predominant in platelets (as opposed to Aß_1-42_ predominant in brain), in various forms of amyloidosis (Inyushin et al.). Platelet-derived Aß has immune functions in infection, where APP processing is non-amyloidogenic. However, this changes in amyloidosis disorders, when systemic Aß contributes to vascular damage. The consensus is that the platelet shift in APP processing to Aß represents an excellent model to study blood-based AD biomarkers. In contrast, evidence also suggests a complex picture whereby changes in platelet components are incompletely replicated in plasma. This is the case for brain-derived neurotrophic factor (BDNF) and its precursor proBDNF, where the cerebrospinal fluid proBDNF/BDNF ratio is a candidate AD biomarker. Plasma and platelets also contain proBDNF and BDNF, but studies from the Lordkipanidze group (Le Blanc et al.; Fleury et al.) show that unlike BDNF, proBDNF is not released from platelets upon activation showing a different proBDNF/BDNF regulation between CNS and plasma.

With similar objectives in mind, Humpel’s group (Foidl et al.) used a lipidomic approach to profile the lipid expression pattern in a murine model of sporadic cerebral amyloid angiopathy (CAA), a vascular pathology which occurs independently, or as a frequent AD co-morbidity. CAA diagnosis relies on vascular deposition of Aß_1-40_. Alterations in lipid profiles in both platelets and plasma (6 platelet lipids and 15 plasma lipids) were identified in the CAA model, which does not exhibit AD pathology, but with differential signatures. Given the difficulty of diagnosing pure CAA, the identification of a unique lipid profile in this disorder may lead to earlier differentiation between CAA and AD.

The potential of classical platelet parameters such as mean platelet volume, platelet count and platelet distribution width as early disease markers is also being explored. Gialluisi’s group (Tirozzi et al.) identified a significant genetic correlation between platelet distribution width and PD risk, but not between AD and platelet parameters. Given that platelet distribution width is an index of platelet procoagulant activity, this parameter may represent a risk indicator for certain neurodegenerative/neuropsychiatric disorders.

The Langer group describes the interplay between platelets and the complement system as well as plasmatic coagulation factors and the potential clinical benefit of targeting platelet-mediated neuroinflammation as an adjunct therapy to mitigate disease severity in MS and stroke-associated brain injury (Rawish et al.). Such developments prompted the design of an MS study by Koudriavtseva and colleagues seeking to establish a link between the pathogenetic role of coagulation and hemodynamic abnormalities in MS. This study aims to correlate magnetic resonance imaging-identified brain hemodynamic changes with altered coagulation/complement factor profiles and related damage markers, with the long-term goal of validating the coagulation system as a therapeutic target in MS (Koudriavtseva et al.).

Concurrently, as described by Orian and collaborators, avenues for platelet imaging and targeting are being explored. The platelet-specific GPIIb/IIIa receptor undergoes conformational changes during activation, thereby exposing a ligand binding pocket enabling differential targeting of the activated counterpart, but not resting platelets. Since activated platelets accumulate at the site of injury, platelet imaging when combined with other imaging approaches may provide improved sensitivity for longitudinal monitoring and candidate therapeutic evaluation. The concept of platelet targeting for therapeutic ends has been hampered by the risk of bleeding complications, but refined targeting of activation-specific epitopes warrants further investigation.

While the link between platelets and neuropathologies is strengthening, similar revelations are being made in other fields. Studies have highlighted the cross-talk between platelets and cancer cells and the role of platelets in tumor metastasis (13, 14). Work in cerebral malaria has shown that both platelets and platelet-derived EV contribute to pathology (15). Therefore, advances in determining the potential of platelets in diagnosis, patient monitoring and as therapeutic targets, would benefit from improved understanding of shared mechanisms across conditions where platelets drive pathological progression and of platelet interaction with their target organs over disease evolution.

## Author Contributions

JMO wrote the first complete draft of the article. SCW and CH contributed to the final draft and edited the article. All authors contributed to the article and approved the submitted version.

## Funding

SCW was funded by the UK Medical Research Foundation (award number MR/S009450/1) and the US National Institutes of Health (award number R21AI142472). JMO was funded by the La Trobe University Research Focus Area scheme, La Trobe Alumni and Multiple Sclerosis Research Australia.

## Conflict of Interest

The authors declare that the research was conducted in the absence of any commercial or financial relationships that could be construed as a potential conflict of interest.

## Publisher’s Note

All claims expressed in this article are solely those of the authors and do not necessarily represent those of their affiliated organizations, or those of the publisher, the editors and the reviewers. Any product that may be evaluated in this article, or claim that may be made by its manufacturer, is not guaranteed or endorsed by the publisher.
